# The Invasive Brazilian Pepper Tree (*Schinus terebinthifolius*) Is Colonized by a Root Microbiome Enriched With Alphaproteobacteria and Unclassified Spartobacteria

**DOI:** 10.3389/fmicb.2018.00876

**Published:** 2018-05-03

**Authors:** Karim Dawkins, Nwadiuto Esiobu

**Affiliations:** Microbial Biotech Lab, Biological Sciences Department, Charles E. Schmidt College of Science, Florida Atlantic University, Boca Raton, FL, United States

**Keywords:** Brazilian pepper tree, Spartobacteria, rhizosphere, microbiome, invasive plant, biotic resistance

## Abstract

Little is known about the rhizosphere microbiome of the Brazilian pepper tree (BP) – a noxious category 1 invasive plant inducing an enormous economic and ecological toll in Florida. Some invasive plants have been shown to drastically change the soil microbiome compared to other native plants. The rhizobacteria community structure of BP, two Florida native plants (*Hamelia patens* and *Bidens alba*) and bulk soils were characterized across six geographical sites. Although all 19 well-known and 10 poorly described phyla were observed in all plant rhizospheres, BP contained the least total bacterial abundance (OTUs) with a distinct bacteria community structure and clustering patterns differing significantly (pCOA and PERMANOVA) from the natives and bulk soil. The BP rhizosphere community contained the highest overall Proteobacteria diversity (Shannon’s diversity 3.25) in spite of a twofold reduction in richness of the Gammaproteobacteria. Remarkably, the invasive BP rhizosphere was highly enriched with Alphaproteobacteria, dominated by Rhizobiales, including Rhodoplanes and Bradyrhizobiaceae. Also, the relative abundance of Spartobacteria under BP rhizosphere was more than twice that of native plants and bulk soil; featuring unique members of the family Chthoniobacteraceae (DA101 genus). The trend was different for the family Pedosphaerae in the phylum Verrucomicrobia where the abundance declined under BP (26%) compared to (33–66%) for the *H. patens* native plant and bulk soil. BP shared the lowest number of unique phylotypes with bulk soil (146) compared to the other native plants with bulk soil (*B. alba* – 222, *H. patens* – 520) suggestive of its capacity to overcome biotic resistance. Although there were no specific biomarkers found, taken together, our data suggests that the occurrence of key bacteria groups across multiple taxonomic ranks provides a somewhat consistent profile of the invasive BP rhizo-community. Furthermore, based on the observed prevalence of a bacteria group (Spartobacteria – Chthoniobacteraceae – DA101); we propose that they have a possible role in BP biology. Our results emphasize the need to further investigate the potential value of “unique phylotypes” in the rhizosphere relative to bulk soil as an ecological tool for monitoring plant-cover/invasion history; or even detecting exotic plants with invasion tendencies.

## Introduction

The Brazilian pepper tree (BP – *Schinus terebinthifolius*) is one of the most tenacious, difficult-to-control invasive plants in the Everglades National Park (ENP), exerting devastating ecological and economic impacts in the area. In the Florida Everglades, BP is the most widely distributed invasive plant, covering over 30,000 ha, followed by Melaleuca (17,802 ha), and Old World climbing fern – 7,033 ha (Rodgers et al., 2014). With more than 283,000 hectares of south and central Florida dominated by this category one invasive plant (Rodgers et al., 2014), current extensive and expensive control/restoration measures, relying on chemical treatment or mechanical uprooting have been minimally successful (Dawkins and Esiobu, 2016). Field managers in Florida continue to face enormous challenges with the fast regrowth of cut stumps, the effective establishment of BP even among natural undisturbed habitats, and the efficiency of the spread of its numerous seeds by frugivorous birds and wind. Unlike Melaleuca (*Melaleuca quinquenervia*), where current control efforts have been successful in restricting spread, the BP is not only resistant but has expanded its range to all parts of the Florida Peninsula (Ferriter, 1997). Worse still, the restoration of native ecology after removal of BP is impeded by the poorly understood BP legacy effect, where soils fail to support the native flora and fauna as expected (Nickerson and Flory, 2015).

Pioneering research to understand the below-ground microbial aspects of BP invasion suggests that enhanced mycorrhizal associations and enemy release phenomena play a role in the successful establishment of BP (Dawkins and Esiobu, 2017). The enemy release mechanism of plant invasion attributes the rapid increase in abundance of a plant species introduced into an exotic region to the escape from its natural enemies in its native region (Keane and Crawley, 2002). In a recent study on the fungal microbiome of BP rhizosphere, it was shown that BP was associated with higher numbers of potentially beneficial arbuscular and ecto-mycorrhiza while harboring few known pathogenic fungi which were found in higher abundance in the rhizosphere of native plants (Dawkins and Esiobu, 2017). This important finding however does not explain the low biotic resistance (Levine et al., 2004) of Florida communities which is often invoked to explain, at least in part, the inability of resident species to resist the success of exotic invaders. Biotic resistance is a measure of the chances that an introduced species would successfully invade the range. It is linked to species richness and diversity, with resident species playing varying roles in resistance to invasive species. Very few studies have addressed the critical microbial component of this phenomenon (Dawkins and Esiobu, 2016). Some invasive plants selectively recruit and associate with specific soil microbiota, alter soil microbial community structure, and exert detrimental effects on native plants, including native seedling failure during restoration efforts (Callaway et al., 2004; Stinson et al., 2006; Perkins and Hatfield, 2016). It is not known whether the resilience of BP in Florida is enhanced by its root microbial symbionts. It is however clear that BP invasion is worst in Florida (Ferriter, 1997; Hight et al., 2003) where two haplotypes (A and B); introduced from separate regions of Brazil hybridized to create a hybrid more invasive than its native counterparts (Williams et al., 2005). It shares well-known invasive mechanisms such as enemy release, adaptations to the physical environment and enhanced nutrient acquisition with other invasive plants (Dawkins and Esiobu, 2016), but the paucity of research on the possible contribution of microorganisms to the biotic resistance or lack thereof in the Florida niche leaves an important piece of the puzzle out. To successfully establish themselves, invasive plants must first overcome biotic resistance which may be in the form of antagonistic soil microbiota and macrobiota as well as other native flora. The rhizosphere microbial community structure of plants is shaped by complex interacting factors including organic exudates such as flavonoids, phenolics, and allelochemicals by plants (Badri et al., 2013); environmental factors such as pH, nutrient levels, and the biogeochemical activities of the microbes themselves (Coats and Rumpho, 2014). These plant–microbe–soil interactions and the resultant microbial community structure in the rhizosphere can determine the abundance and diversity of above ground flora and potentially play a significant role in the success or demise of invasive plants (Reinhart and Callaway, 2006). Some invasive plant species associate with beneficial soil microorganisms in their rhizosphere, which promote plant growth by enabling the uptake of additional nutrients and improving stress tolerance and protection against other pathogenic microorganisms (Berendsen et al., 2012). Invasive plants not only spread across different ecosystems but could also displace native flora by restructuring the root microbiome and disruption of nutrient cycling (Pringle et al., 2009; Rodrigues et al., 2015) along with multiple other invasive mechanisms (Levine et al., 2003; Bennett et al., 2014).

We hypothesized that if the BP rhizobacteria are important for successful establishment in Florida soils, then the BP rhizobiome will select for specific communities regardless of sample source. Using amplicon next generation sequencing of the bacterial V4 region of 16S rDNA, the bacterial rhizosphere community structure of BP and two Florida natives, including bulk soil samples were characterized across six geographical sites in three counties.

## Materials and Methods

### Sampling and Sample Sites

In order to test the hypothesis that BP roots are colonized by a stable and consistent soil bacterial community that potentially contributes to its notorious biology, six sites spanning the South-eastern Florida from Palm Beach, Broward to Miami-Dade counties (**Table 1**) were studied. Factors considered in site selection included a verifiable history of Brazilian pepper invasion, the presence of dominant or monoculture stands of Brazilian pepper, a consistent age of Brazilian pepper plants (> 1 year) and the co-existence of the two Florida native plants in adjacent (∼600 m) parcels of land. The two Florida native species, Shepherd’s needle (*Bidens alba*) and Firebush (*Hamelia patens*) were chosen as the non-invasive plant species whose rhizosphere microbial communities will be compared with that of the BP. Shepherd’s needle is an annual or perennial short-lived herbaceous weed which in a previous study (Morgan and Overholt, 2005) was shown to be inhibited by BP leaf extracts. Firebush is a fast growing perennial shrub plant similar to the BP which is found extensively throughout South-eastern Florida.

**Table 1:** Description of sample site locations across three counties with the 16S rDNA reads, OTU counts (post-normalization) and rhizosphere/bulk soil pH values resolved per sample ID.

Sample Sites	County	SampleID	16S Reads	OTU Counts	pH
Site 1/Tree Tops Park	Broward	ST1	197184	5111	5.62
26° 4′ 18″N, 80° 16′ 35″W	Broward	HP1	80893	6361	7.5
	Broward	BA1	175924	5417	6.9
	Broward	BULK1	299256	5531	7.58
Site 2/Coconut Creek	Broward	ST2	311422	5009	6.44
26° 14′ 5.6″N, 80° 11′ 16″W	Broward	HP2	332272	5448	7.35
	Broward	BA2	245642	4644	6.69
Site 3/West Delray Regional Park	Palm Beach	ST3	168830	4668	5.63
26° 27′ 41″N, 80° 13′ 10.8″W	Palm Beach	HP3	292544	5429	7.44
	Palm Beach	BA3	184227	5526	5.17
	Palm Beach	BULK3	266347	5517	7.29
Site 4/Dyer Park	Palm Beach	ST4	203199	4636	6.56
26° 47′ 19″N, 80° 7′ 22″W	Palm Beach	HP4	165333	5311	7.15
	Palm Beach	BA4	204588	5231	6.74
	Palm Beach	BULK4	197333	3492	6.58
Site 5/R Hardy Matheson Preserve	Miami-Dade	ST5	127781	3752	5.92
25° 39′ 24″N, 80° 16′ 48″W	Miami-Dade	HP5	372824	5454	7.04
	Miami-Dade	BA5	215695	4575	6.36
	Miami-Dade	BULK5	311981	5287	7.04
Site 6 Oleta River State ParkΦ	Miami-Dade	ST6	227118	5589	7.98
25° 55′ 0.3″N, 80° 8′ 19″W	Miami-Dade	HP6	273032	4707	7.58
	Miami-Dade	BULK6	214826	5093	6.94
		STHP	223427	5516	
Totals			5,967,188	16089	

*Φ site observed with the least number of monoculture *Schinus* stands and different soil texture (coarse/sandy) compared to other sites (loamy/sandy).*

**Table 2:** Average values and standard error of OTU counts and pH for each sample type.

Sample Type	OTUs	pH
*Schinus*	4794 ± 252^a^	6.36 ± 0.36^a^
*Hamelia*	5452 ± 216^a^	7.34 ± 0.09^b^
*Bidens*	5079 ± 180^a^	6.37 ± 0.29^a^
Bulk soil	4984 ± 348^a^	7.09 ± 0.15^a^

*Column mean values followed by different letters are significantly different (Tukey HSD <0.05).*

### Sampling Protocol

At each sampling site, three dominant stands of BP (>1 year old) were identified and sampled at a depth of 15–20 cm where roots and adjacent soil were removed. For each BP stand, three rhizosphere samples were collected by digging up roots with surface sterilized shovels and knife; and then shaking off the soil into a sampling bag; to make one composite sample (Rep 1; İnceoğlu et al., 2011). The process was repeated twice to produce three BP replicates for each site, yielding a total of 18 composite BP samples. Each replicate was sampled at least 10 meters apart. In addition, three replicates of rhizospheric soil samples were collected from two Florida native plants – Firebush (*H. patens*) and Shepherd’s needle (*B. alba*) for each location using the same protocol where the native plants and bulk soil were all collected within a ∼600 meter radius. As a control, bulk soil without plant cover was sampled at the same depth using an improvised sterile soil borer of 12 cm length by 3 cm diameter. Three to five centimeters of top soil was first removed to eliminate litter and debris before samples were collected. The number of samples collected was seventy-two (72) [(3 Reps × 3 Plants × 6 sites) + (3 Bulk × 6 Reps)]. An additional sample set was taken from a relatively young *S. terebinthifolius* plant found growing alongside the native *H. patens* located behind the FAU Davie Greenhouse (STHP) following the same sampling protocol for a total of 75 samples. At site 6, there was a campaign to remove BP from the area through use of herbicides and physical removal. The monoculture stands found at this site did not seem to be flourishing very well, and the soil condition was coarse and sandy. After collection, samples were stored at -20°C prior to extraction. DNA from the 75 samples was then extracted separately and the replicates pooled for a final tally of 25 samples.

### Soil Chemical Analysis

Chemical analysis of soil samples was performed at the Florida Atlantic University, Department of Geosciences Water Analysis Lab (Davie, FL, United States) using 10 g of rhizosphere and bulk soil for pH determination. Soil pH was measured using distilled water with a calibrated Sartorius PB-11 pH meter (Sartorius Corp., NY, United States).

### Extraction of Community Genomic DNA From Bacteria

Two grams of total DNA instead of 0.75 g (recommended by the manufacturer) was collected from the rhizosphere of each plant along with bulk soil and extracted using the MoBio Powersoil kit (Mo Bio Laboratories Inc., CA, United States). This modified technique significantly increases the number of cells extracted from the sample the matrix. The two grams of soil samples were vigorously vortexed in 10 ml sterile 1× Phosphate buffer for 10 min to dislodge bacteria cells, which were then harvested into a pellet at 13,000 rpm for 5 min and subjected to the MoBio Powersoil kit according to manufacturer instructions.

### Validation of DNA Quality

The DNA concentration and purity of each sample was measured using the Nanodrop 2000c spectrophotometer (Thermo Fisher Scientific, MA, United States). Extracted samples were also run on a 1% agarose gel (1× TAE) at 90 V for 45 min to determine if DNA bands were intact. To further confirm if the extracted DNA is amplifiable, the bacterial 16S rDNA was amplified using the universal primers 1492 Reverse (5′GGTTACCTTGTTACGACTT-3′) and 27 Forward (5′-AGAGTTTGATCCTGGCTCAG-3′) that targets the V1–V9 region of the 16S rDNA gene and produces an approximate 1500 bp fragment. Reaction mixtures were incubated for 4 min at 94°C for denaturation, followed by 35 cycles consisting of 1 min at 94°C, annealing for 30 s at 45°C, and extension for 2 min at 72°C using Taq Polymerase from the 2× Promega PCR master mix (Promega Corp., WI, United States) along with 0.4 μM of each primer, 10 μg bovine serum albumin (Promega Corp., WI, United States), 2 mM MgCl_2_, and approximately 20 ng of DNA template for each sample in a final 25 μl reaction mix. Four microliters of PCR product were run using gel electrophoresis on a 1% agarose (w/v) gel at 90 V for 45 min to view the expected ∼1500 bp band size.

### Preparation of Extracted (Pooled) Samples for Sequencing

After confirmation of successful amplifications, twenty microliters of DNA from each of three replicates from *S. terebinthifolius, H. patens, B. alba*, and bulk soil at each site were combined into separate single tubes to yield (75/3) 25 different samples with 60 μl of representative genomes. To ascertain the concentration of the 25 pooled extracted DNA samples, the DNA was first quantified using the Qubit 2.0 fluorimeter (Thermo Fisher Scientific Inc., MA, United States) according to manufacturer recommendations and the Illumina sequencing protocol.

### PCR Amplification With Illumina Primers 515F/806R

A second validation was done on the normalized pooled DNA samples to ensure that they are able to be amplified using the Illumina recommended primers which have been used in another study to study the soil microbiome (Bergmann et al., 2011). The selected bacterial/archaeal primers 515F (5′-AATGATACGGCGACCACCGAGATCTACAC TATGGTAATT GT GTGCCAGCMGCCGCGGTAA-3′)/806R (5′-CAAGCAGAAGACGGCATACGAGAT AGTCAGTCAG CC GGACTACHVGGGTWTCTAAT-3′) were used to target the V3/V4 region of the 16S rDNA sequence for the Illumina MiSeq platform. A further validation was carried out on extracted samples using the 515F/806R primers (0.2 μM each) as shown above. The 5 Prime hot-start 2× master mix (5 PRIME Inc., MD, United States; item #2200410) was used to produce a 25-μl total reaction volume along with PCR grade water and 5 ng of DNA template. The thermocycler conditions used were 94°C for 3 min, 35 cycles of 94°C for 45 s, 50°C for 1 min, and 72°C for 90 s, followed by a subsequent 10 min at 72°C. Two samples did not amplify successfully (bulk soil site 2 and *B. alba* site 6). Four microliters of the amplified products (approximately 300 bp) were run on a 1.5% agarose gel (0.5% TAE) at 70 V for 45 min to validate the amplicon size using a 100 bp ladder.

### Tagging of Individually Amplified DNA Samples for Sequencing

Since only 23 of 25 samples were amplifiable, 23 unique 806R barcoded primers were used to amplify these remaining samples. This second PCR involved the use of the 515F (as shown above) and 806R barcoded primer (5′-CAAGCAGAAGACGGCATACGAGAT XXXXXX AGTCAGTCAG CC GGACTACHVGGGTWTCTAAT-3′) expected to produce a ∼385 bp product. This barcoded PCR was performed as described previously. Barcoded-PCR samples were cleaned using the Agencourt AMPure XP beads (Beckman Coulter, Inc., CA, United States) according to manufacturer’s instructions. The bioanalyzer or Agilent 2200 Tapestation system (Agilent Technologies Inc., CA, United States) was used to validate and quantify the expected base pair size of the amplified fragments (approximately 385 bp).

### Final Library Preparations

After further dilutions and denaturation of DNA samples and the supplied PhiX control, a custom Read 1 (5′-TATGGTAATT GT GTGCCAGCMGCCGCGGTAA-3′), Read 2 (5′-AGTCAGTCAG CC GGACTACHVGGGTWTCTAAT-3′) and index primers (5′-ATTAGAWACCCBDGTAGTCC GG CTGACTGACT-3′) were added to the MiSeq cartridge prior to loading. These primers are expected to amplify the forward and reverse reads (approximately 250 bp) along with the index barcode (6 bp) from each barcoded DNA sample. The sequencing primers along with the pooled sample and PhiX control were aliquoted into separate wells of the MiSeq loading cartridge according to manufacturer instructions.

### Processing of Sequence Data Using QIIME, R, and CosmosID Platform

The forward and reverse reads were paired using the join_paired_ends.py script in QIIME 1.8 (Quantitative Insights in Microbial Ecology; Caporaso et al., 2010). Joined paired-end reads were then quality filtered using the q30 standard where the probability of an incorrect base call is 99.9%. Sequences that did not fit these criteria were removed. A second quality control step – chimera check (identify_chimeric_seqs.py) was done to identify and remove chimeric sequences. Demultiplexing and quality filtering were done using the “split_libraries_fastq.py” script and a quality filtering score set at phred 30. The operational taxonomic unit (OTU) table in “biom” format was then generated by picking OTUs (97% sequence similarity) using the “pick-reference-otus.py” script that were aligned against the Greengenes database (DeSantis et al., 2006) for 16S rDNA. Singleton sequences were removed, and each sample was subsampled to 80893, which was the minimum number of sequences remaining in a single sample. Additional analysis of the OTU tables using alpha and beta diversity measures along with a taxonomy summary indicating the relative abundances for each sample was performed using heatmaps from CosmosID. Alpha and Beta analyses were generated by using the QIIME 1.8 and R software (Caporaso et al., 2010; R Core Team, 2017). Shannon’s alpha diversity index was computed using the vegan R package based on OTU counts (Oksanen et al., 2016). Heatmaps were prepared based on the relative abundance of the OTU counts and clustered using the Euclidean distance and Ward clustering algorithm (Murtagh and Legendre, 2014). The Venn diagram plots and centroid plots were created using OTU data in R package “gplots” (Warnes et al., 2016) and relative abundance data in “ggplots2” (Wickham, 2009) respectively. The STHP sample from a much younger *S. terebinthifolius* plant growing alongside the *H. patens* native will only be used to assess the relative abundance, diversity, and richness of the different bacterial taxa expressed in heatmaps and the pCOA plot.

All sequences were deposited in the NCBI BioSample database with accession numbers SAMN06210629–SAMN06210651 and Bioproject ID 338194.

### Statistical Analysis

The statistical significance of the OTUs, Shannon’s diversity index, and observed relative abundance and diversity measures was tested using two-way ANOVAs at the 95% confidence limit followed by the *post hoc* Tukey HSD test if significance was found The Kruskal–Wallis non-parametric test was used for data that didn’t fall within equal variance. Bartlett’s test was used to verify normality of the data. To assess the correlation of the relative abundances of the different bacterial taxa with measured abiotic factors, the Pearson correlation coefficient was used.

## Results

### 16S Operational Taxonomic Units (OTUs)

The bacterial community structure of BP in its invasive range was evaluated across six geographic sites using the total genomic DNA from the rhizosphere (*n* = 6). Likewise, bacterial community structure of a non-phylogenetically related Florida native shrub, *H. patens* (*n* = 6) and a common weed, *B. alba* (*n* = 5), and bulk soil samples (*n* = 5) were similarly evaluated. A total of 5,967,188 sequenced reads was obtained after the initial quality filtering at Q30 (99.9% base call accuracy). Operational taxonomic units (OTUs) were generated using an open reference allocation where sequences with >97% similarity was grouped as a single OTU using the UCLUST algorithm. Of the total OTUs obtained (16,089) for all the samples (**Table 1**), *S. terebinthifolius* had the lowest average number of OTUs (4,794 +/-252) while *H. patens* native had the highest average number of OTUs (5,451 +/-216). The *B. alba* native and bulk soil registered similar OTU averages of 5,079 +/-180 and 4,984 +/-348, respectively. A one-way ANOVA of OTU counts for the three different plant types revealed no significant difference between them (*p* = 0.14) at the 95% confidence level. Unpaired *t*-tests between *S. terebinthifolius* and bulk soil also showed no significant difference between the OTU means (*p* = 0.67).

### Prevalence and Relative Abundance of Specific Bacteria Taxa

**FIGURE 1:**
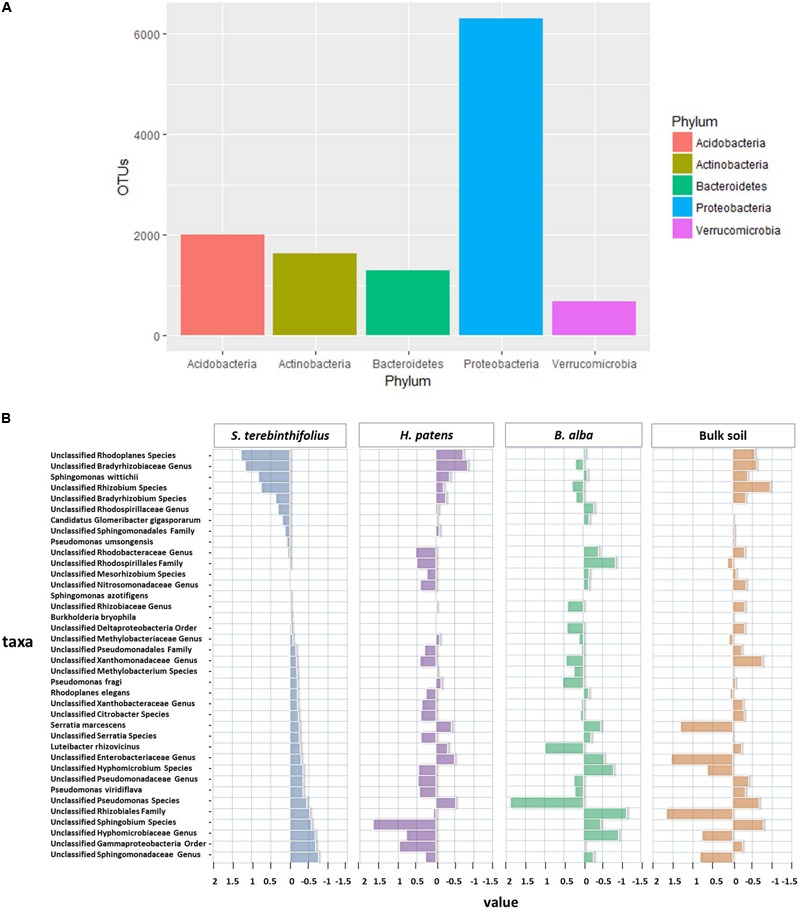
**(A)** Overall relative abundance of top five phyla observed throughout all plant types and bulk soil. **(B)** The distances from the average relative abundance of Proteobacteria taxa for each plant type (invasive *S. terebinthifolius*, native *H. patens*, and native *B. alba*) and bulk soil where positive values have a relative abundance higher than the average and negative values have a relative abundance lower than the average. *S. terebinthifolius* has higher relative abundances of members of the Alphaproteobacteria class including unclassified *Rhodoplanes*, Bradyrhizobiaceae, and *Rhizobium* species.

A total of 19 known and 10 poorly described bacterial phyla (Youssef et al., 2015) were identified in this study. The top phylum observed in all locations was the Proteobacteria which amounted to 6304 OTUs (39.2%). The *Acidobacteria* and *Actinobacteria* registered 1999 OTUs (12.4%) and 1637 (10.2%) OTUs, respectively. *Bacteroidetes* and *Verrucomicrobia* rounded out the top 5 with 1290 (8%) and 687 (4.2%) OTUs, respectively (**Figure 1A**).

### Rhizosphere Bacterial Community Structure of the Brazilian Pepper Tree

The top five phyla most abundant under BP rhizosphere included Proteobacteria (47.7%), Acidobacteria (14.5%), Bacteroidetes (9.6%), Verrucomicrobia (9.3%), and Actinobacteria (7.7%). Although the rhizosphere of all the plants and bulk soil were dominated by the phylum Proteobacteria; it was relatively reduced under the BP rhizosphere (47.7% of community) compared to *H. patens* (52.1%) and *B. alba* and bulk soil with 64.3% and 63.7% respectively. At the class level, BP rhizosphere was associated with Gammaproteobacteria, Alphaproteobacteria, Deltaproteobacteria, and Betaproteobacteria at 25%, 12.8%, 5.8%, and 5.7% relative abundance respectively. In the Gammaproteobacteria class, the community structure shift was most striking under BP rhizosphere compared to the other native plants and bulk soil as shown by a twofold reduction in relative abundance (25%) compared to bulk soil (58.8%) with *p* = 0.047 using a one-way ANOVA (3, 13) = 7.82 and confirmed by Tukey HSD. The key bacterial order that was reduced was the Enterobacteriales, especially the *Serratia* spp. group which had a 19% relative abundance across all sites for *S. terebinthifolius* compared to a 22% and 58% relative abundance associated with the *H. patens* native and bulk soil respectively seen as potential variation (**Figure 1B**). In the Alphaproteobacteria class, *S. terebinthifolius* had a significantly higher proportion of the Rhizobiales (14.2%) than the native plant *B. alba* – 7.2% using a two way ANOVA (3,13) = 7.56, *p* = 0.0296, and confirmed using the Tukey HSD *post hoc* test (Supplementary Table S1).

**FIGURE 2:**
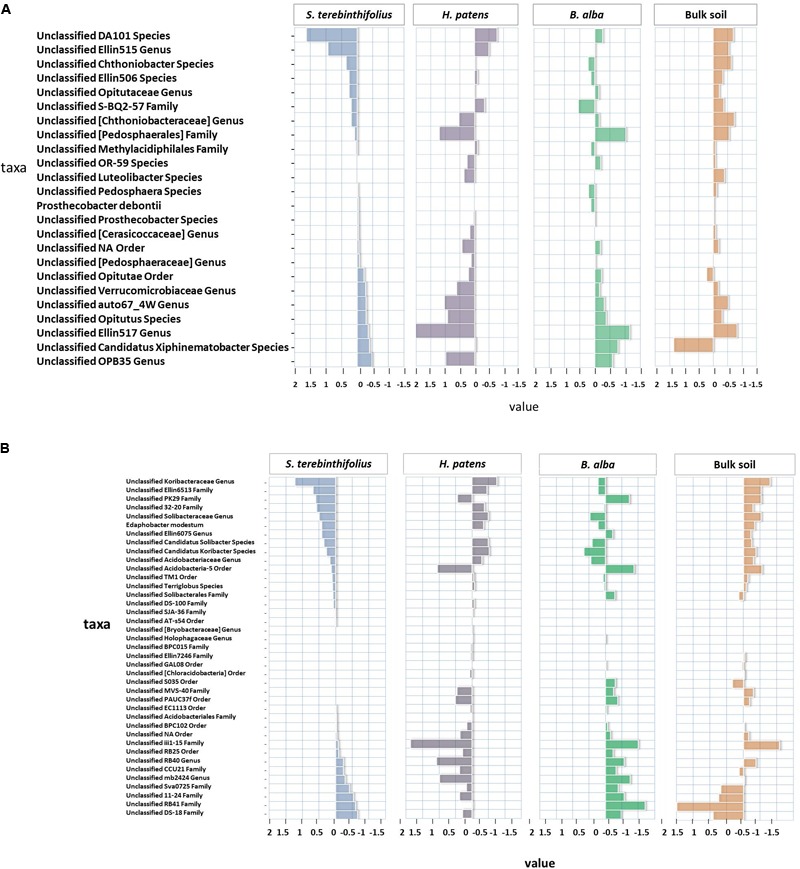
**(A)** The distances from the average relative abundance of Verrucomicrobia taxa for each plant type (invasive *S. terebinthifolius*, native *H. patens*, and native *B. alba*) and bulk soil where positive values have a relative abundance higher than the average and negative values have a relative abundance lower than the average. The DA101 and Ellin515 unclassified Genus was more prevalent under *S. terebinthifolius* than the other native plants and bulk soil. **(B)** The distances from the average relative abundance of Acidobacteria taxa for each plant type (invasive *S. terebinthifolius*, native *H. patens*, and native *B. alba*) and bulk soil where positive values have a relative abundance higher than the average and negative values have a relative abundance lower than the average. In the rhizosphere of *S. terebinthifolius* the unclassified genus Koribacteraceae and Ellin6513 family are found predominantly compared to the native plants and bulk soil.

The most prevalent taxa of the Rhizobiales were from the families Hyphomicrobiaceae and Bradyrhizobiaceae dominated by the unclassified Rhodoplanes and Bradyrhizobiaceae, respectively (**Figure 1B**). The most obvious changes in the Proteobacteria community structure for BP occurred between the Alpha and Gammaproteobacteria class where a subsequent reduction in Gammaproteobacteria (25%) coincided with an increase in the abundance of Alphaproteobacteria -27.8%. In the phylum Acidobacteria, there was an average relative abundance of 16.3% across all BP sampling sites with sample site 2 recording the highest relative abundance (23.5%) and bulk soil recording 10.8%. An unclassified Koribacteraceae and Ellin6513 at the family level was most prevalent in the Acidobacteriia class and stood out as unique taxa for *S. terebinthifolius* (**Figure 2B**) where the centroid plot shows that the average relative abundance of both taxa was high under BP but were not well represented under the other plant types and bulk soil.

Bacteroidetes, the third most abundant phylum under BP was triple (9.6%) that of the relative abundance of bulk soil (3%). The four most dominant orders were Saprospirales, Sphingobacteriales, Cytophagales, and Flavobacteriales. Saprospirales was the most prevalent order and its highest abundance was recorded for bulk soil (66% of Bacteroidetes) followed closely by 62% of Bacteroidetes for BP rhizosphere. BP shared similar relative abundances of Sphingobacteriales, Cytophagales, and Flavobacteriales with the native plant types and bulk soil. Quite remarkably, of the top five bacteria phyla recorded in this study, Verrucomicrobia was one of the most abundant in the rhizosphere of the invasive *S. terebinthifolius* plants (9.2%) with a near twofold increase in occurrence compared to the native plants *B. alba* (3.8%) and *H. patens* (4.4%). These differences were found to be significant for both native plants and bulk soil at the 95% level in this study [ANOVA – *F*(2,14) = 9.7, *p* = 0.00185 – confirmed by the Tukey HSD test; Supplementary Table S2].

In fact, the native plants mirrored the prevalence rate of the Florida bulk soil which recorded the lowest Verrucomicrobia relative abundance at 2.4% of community. Spartobacteria was the most dominant class from Verrucomicrobia reported for the invasive plant *S. terebinthifolius* where the unclassified Genus DA101 and Ellin515 from the family Chthoniobacteraceae were most prevalent (**Figure 2A**) and were found in substantially lower numbers in the other native plants and bulk soil. It is noteworthy that the elevation in the abundance of the Chthoniobacteraceae family under BP coincided with a drop in numbers for the family Pedosphaerae in the Spartobacteria class.

Only 26% +/6.8% of Verrucomicrobia relative abundance was represented by the Pedosphaerae family under BP rhizosphere whereas it was elevated to 33–67% in the population of Florida native plants and bulk soil. Results for BP at site 6 were the main outlier, showing a Pedosphaerae family population level similar to natives at 56% of Verrucomicrobia relative abundance. The Pedosphaerae family was significantly reduced under BP rhizosphere compared to the natives (*p* = 0.0018) using the Kruskal–Wallis test. The fifth most prevalent phylum in the BP rhizosphere, Actinobacteria, had a relative abundance of 7.7%. The most abundant class under the Actinobacteria phylum was Thermoleophila which represented ∼50% of all Actinobacteria reads. Solirubrobacterales order and unclassified Solirubrobacterales family was most prevalent in the Thermoleophila class. The Actinobacteria class had the second highest relative abundance under the phylum and was consistently represented at each site for BP. At the order level, BP site 6 was different; showing an elevated relative abundance of the Acidimicrobiales order (26% of Actinobacteria) compared to the other sites that had an average of 10% of Actinobacteria for these bacteria.

### Bacterial Community Structure of Native Plants and Bulk Soil

Both native plants shared the same top five most dominant phyla as BP but had Verrucomicrobia as least dominant where there was a two- to threefold reduction for *H. patens* (4.5%), *B. alba* (3.9%), and bulk soil (2.4%) compared to BP (9.3%). In the Spartobacteria class, the abundance of the Chthoniobacteraceae family was relatively low under *H. patens* (25%), *B. alba* (59%), and bulk soil (45%) but had an elevated relative abundance for the Pedosphaerae family – *H. patens* (66.5%), *B. alba* (33%), and bulk soil (47.8%). The Proteobacteria phylum relative abundance was elevated under *H. patens* (52.1%), *B. alba* (64.3%) and bulk soil (63.7%). The native plants and bulk soil were dominated again by the Gammaproteobacteria. *B. alba* had the same relative abundance of Enterobacteriales as BP but had a higher relative abundance of Pseudomonadales (40%) where *Pseudomonas* spp. was most prevalent; and was the most distinct characteristic for the *B. alba* native. For the Acidobacteria phylum, native plants, *H. patens, B. alba*, and bulk soil had relative abundances of 15.5%, 10.5%, and 11.3% respectively. The relative abundance of the Bacteroidetes phylum under *H. patens* and *B. alba* was 6.5% and 6% respectively with 2.9% reported for bulk soil. The four most dominant orders were Saprospirales, Sphingobacteriales, Cytophagales, and Flavobacteriales.

### Abiotic Parameters at Study Sites

**Table 3** displays the metadata from the sample sites, including the average rainfall and temperature during the sample collection months as well as the soil type, BP invasion history, and key geographical features.

**Table 3:** Additional metadata from sampling sites: rainfall precipitations, temperature, Brazilian pepper tree invasion history, soil type, and any key features.

Sites	County	Sample date	Rainfall/inches	Avg. temp. /degrees C	BP invasion history	Soil type	Key features
Site 1 Tree Tops Park	Broward	3/21/2015	0	25.5	>15 years	sandy/loamy	multiple dominant stands
Site 2 W Atlantic Ave/Lyons Rd.	Broward	3/28/2015	0.27	25.5	Unknown	sandy/loamy	multiple dominant stands
Site 3 West Delray Regional Park	Palm Beach	4/4/2015	0	24.4	>15 years	sandy/loamy	few dominant stands with a lake close by
Site 4 Dyer Park	Palm Beach	4/11/2015	0	27.7	>15 years	sandy/loamy	few dominant stands with a lake close by
Site 5 R Hardy Matheson Preserve	Miami-Dade	4/18/2015	0	25.5	>15 years	sandy/loamy	few dominant stands
Site 6 Oleta River State Park	Miami-Dade	5/7/2015	0	25.5	>15 years	coarse sandy	very few dominant stands

Site 6 was different; it had a coarse/sandy soil texture, the smallest population of BP monoculture stands, and the highest average soil pH. Compared to other plants and bulk soil, BP rhizosophere soil had the lowest average pH and was significantly different from the average pH under the native *H. patens* using ANOVA (3,13) = 3.9, *p* = 0.034 confirmed by the Tukey HSD *post hoc* test (**Tables 1, 2**).

Spearman’s rank correlation was used to assess the effects of the soil chemistry parameter pH on the relative abundance of the different taxa in the rhizosphere of S. *terebinthifolius*. When comparing the rhizosphere pH of *S. terebinthifolius* from the six sites to the bacterial relative abundance using Spearman’s rank correlation, it was revealed that only Acidobacteriales and Burkholderiales were significantly negatively correlated with soil pH with r value of -0.77 and *p* values of 0.02 at the 95% confidence limit.

### Alpha and Beta Diversity and Cluster Analysis

**FIGURE 3:**
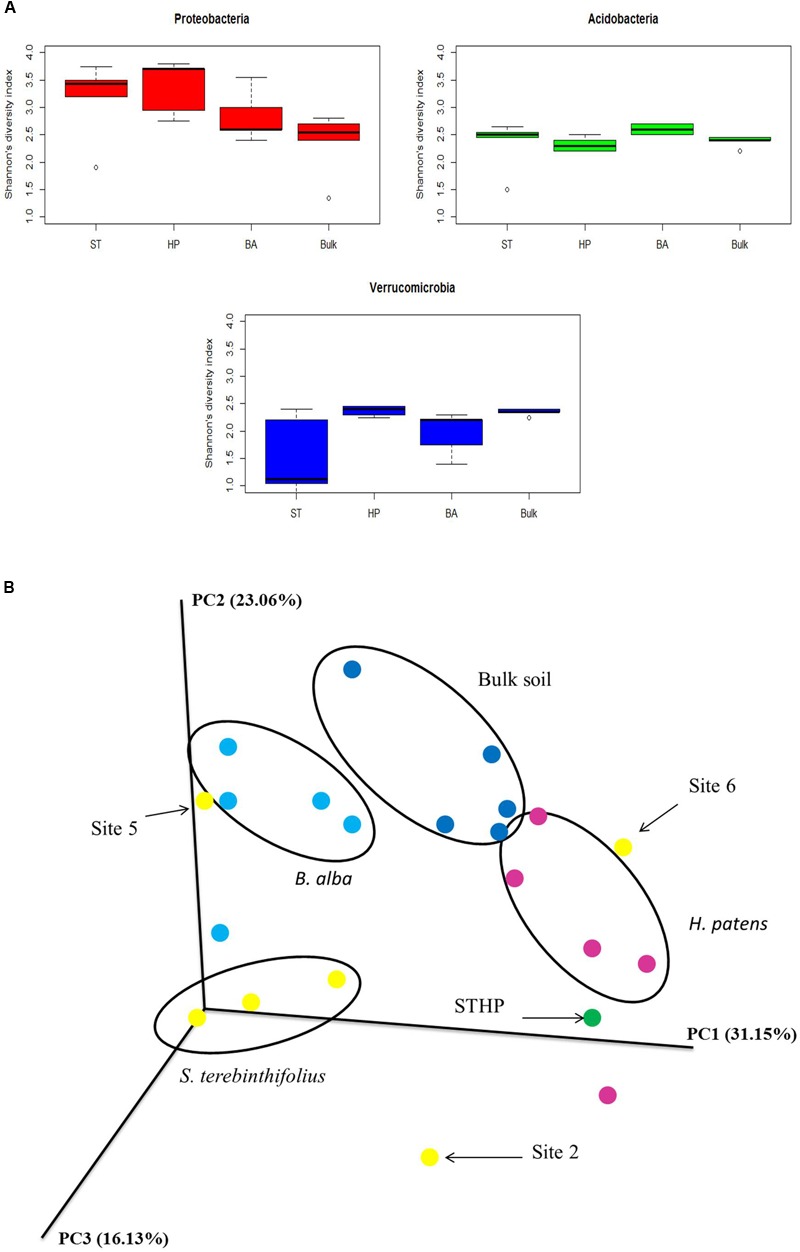
**(A)** Boxplots of phylum-specific OTU alpha diversity (Shannon’s index) from three phyla most dominant under the different plant types (*S. terebinthifolius* – ST, *B. alba* – BA, *H. patens* – HP) and bulk soil across the six sites. Shannon’s index computed using OTU counts in the vegan R package. **(B)** Beta-diversity pCoA weighted UniFrac plot of the different plant types and sites showing the community structure relationships of *S. terebinthifolius, B. alba, H. patens*, and bulk soil. Sites 2, 5, and 6 from *S. terebinthifolius* did not cluster together.

The most distinct changes in soil community structure for BP occurred in three phyla, Proteobacteria, Acidobacteria, and Verrucomicrobia. The average Shannon’s diversity index for the different plant types and phyla (Proteobacteria, Acidobacteria, and Verrucomicrobia) ranged from 0.8 to 3.9 (**Figure 3A**). *H. patens* and BP had the highest Proteobacteria diversity (3.4) but BP had the lowest richness (4794; **Table 2**). The Shannon’s diversity index at the phylum level for Proteobacteria was expectedly lowest under bulk soil devoid of plants (2.4). A significant difference was only found between the different plant types and bulk soil using a one-way ANOVA with *p* value 0.0056, confirmed by Tukey HSD *post hoc* test. Similarly, only BP (2.6) and *H. patens* native plant (2.2) had a significant difference in the Acidobacteria diversity with a *p* value of 0.0056 using the Kruskal–Wallis non-parametric test. Although BP rhizosphere had a lower Shannon’s diversity of Verrucomicrobia than the native plants and bulk soil, the phylum population density was elevated under BP with the highest relative abundance and richness. Verrucomicrobia Shannon’s diversity for BP (1.8) was only significantly different than bulk soil (2.35) and the *H. patens* native (2.4) using the Kruskal–Wallis non-parametric test with *p* value 0.01, confirmed by the Dunn’s *post hoc* test.

Cluster analysis of all the sequence data is shown in **Figure 3B**. From the pCOA plot, only 50% of *S. terebinthifolius* sites clustered together while more than 80% of the native plants were grouped together. *S. terebinthifolius* and *H. patens* growing alongside each other (STHP; green, **Figure 3B**) did not cluster with either monoculture of *S. terebinthifolius* or *H. patens* samples in the pCOA plot, having its own unique soil community structure. Overall, there was a significant difference between the plant type communities (PERMANOVA, *F* = 3.6, *p* = 0.001) and a significant difference between BP and both native plant communities (*p* = 0.023, *p* = 0.021) showing that the different species harbored their own unique bacterial community structure.

### Shared Phylotypes Among the Invasive Plant, Florida Soils, and Native Plants

**FIGURE 4:**
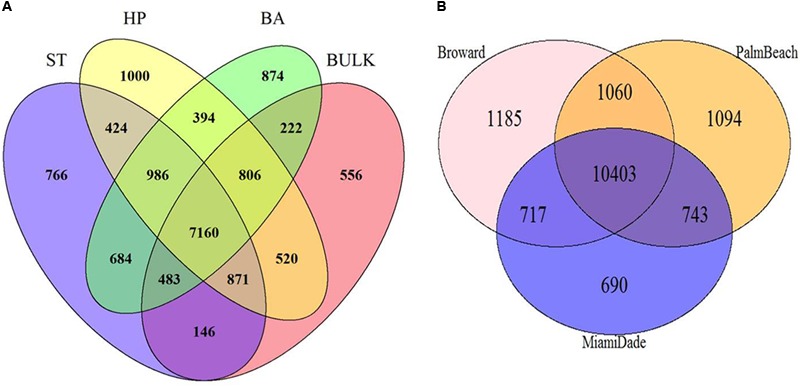
Number of shared phylotypes observed between **(A)** plant types [*S. terebinthifolius* – ST (*n* = 6), *H. patens* – HP (*n* = 6), *B. alba* – BA (*n* = 5), and bulk soil (*n* = 5)] and **(B)** three South Florida counties across the six sampling sites.

A total of 7,160 phylotypes were shared between all plant types and bulk soils (**Figure 4A**) while the Florida native *H. patens* had the most unique phylotypes not found in the other plant types. BP had the lowest number of unique phylotypes in its rhizosphere (766) while *B. alba* and *H. patens* had 874 and 1000, respectively. BP was revealed to have the lowest number of unique phylotypes (146) shared with bulk soil. Broward county had the highest number of unique phylotypes (1185) where Miami-Dade county had the lowest (690) (**Figure 4B**). Both Broward and Palm-beach counties shared less phylotypes with Miami-Dade county.

## Discussion

The rhizosphere microbial community structure is an important determinant of the vegetation and overall plant cover (Reinhart and Callaway, 2006; Berendsen et al., 2012). It is a dynamic entity whose composition is defined by a complex interaction between available microbiota (indigenous or exotic), plant type (including genotypes), soil, and climatic conditions. We designed this study to define, perhaps for the first time, the rhizosphere bacteria community structure of the invasive *S. terebinthifolius* using the adequately robust 16S amplicon sequencing (White et al., 2017). We also seek to define the nature of the differences and to determine whether studying the underlying causes of these community shifts as well as their implications in plant invasion is warranted. It is quite possible that certain invasive plants are capable of releasing exudates under appropriate conditions that will elicit selective or enrichment pressure on soil microbes that eventually favor their survival at the expense of other plants. Other invasive species such as *Centaurea diffusa* and *Centaurea solstitialis* (Callaway et al., 2004; Batten et al., 2006) drastically change the soil microbial structure during spread. Our observations and the latter references underscore the need to understand what triggers this type of microbial community manipulation by plants in their non-native range and not in the indigenous ecology. Multiple locations with history of aggressive displacement of native plants were sampled to determine whether reproducible phylotypes, potentially associated with the invasive attributes could be detected. In parallel, the community profile of two Florida native plants and the corresponding bulk soils in the same locations were studied to contrast the niches with the Florida natural soils and among and between the plants.

The total number of reads obtained (5,967,188) using the Illumina MiSeq is comparable to that acquired in other microbiome studies (Teixeira et al., 2010; İnceoğlu et al., 2011). This study confirmed that the rhizosphere of plants generally contain a greater diversity of bacteria than adjacent bulk soils and more profoundly in the Proteobacteria phylum. Shannon’s diversity index at the phylum level for Proteobacteria, for example, was lowest in bulk soil devoid of plants (**Figure 3A**). The plant rhizosphere is more diverse than bulk soil due to the numerous nutrient exudates and signaling molecules produced by the plant roots (Lagos et al., 2015) as well as other root-associated benefits like predation shield, mycorrhizal advantages, and increased attachment niches.

Operational taxonomic units were picked based on a 97% similarity between sample reads. The OTU richness generally does not give a full picture of the bacterial community structure. It just indicates the number of bacteria present without taking into account their relative abundance and potentially duplicated function/niche; the differences between the BP and the Florida native plants are noteworthy. Curiously for the BP, the Shannon diversity index of Proteobacteria was high (3.4) but the OTU richness low indicative of a more evenly distributed community with a reduced number of species compared to other plant types. Members of the phylum Proteobacteria have been reported as one of the most dominant types of bacteria present in the soil rhizosphere and adjacent soil due to their ability to reproduce quickly and efficiently using plant-derived carbon sources (Janssen, 2006; Lagos et al., 2015). This notion was confirmed in this study as Proteobacteria was the most dominant phylum recovered with an average of 52% relative abundance regardless of plant type and location (**Figure 1A**). The second and third most dominant phyla, *Acidobacteria* and *Bacteroidetes*, were also known to be common components of the soil rhizosphere where the phylum *Bacteroidetes* has many members of the plant growth promoting (PGP) bacteria (Janssen, 2006). One distinction of the BP rhizobiome structure at the phylum level lies in the significantly lower relative abundance of the phylum Proteobacteria (vis à vis other members of BP “rhizocommunity”), compared to the *B. alba* and *H. patens* native plant. So, while the overall Shannon diversity was high for BP’s Proteobacteria, it contained a varied relative abundance distribution of the main four classes – Gammaproteobacteria, Alphaproteobacteria, Betaproteobacteria, and Deltaproteobacteria. Under BP rhizosphere, the Gammaproteobacteria relative abundance was drastically reduced compared to bulk soil while that of the Alphaproteobacteria increased proportionately, with the exception of BP at sites 5 and 6. The major order from Gammaproteobacteria present was the *Enterobacteriales* which consists of Gram-negative, mostly motile facultative anaerobes that serve beneficial or pathogenic roles. The most abundant species found was *Serratia* spp. which could serve as plant pathogens or plant growth promoting (PGP) bacteria depending on the strain and plant (Lavania et al., 2006). A previous study (Dawkins and Esiobu, 2017) has shown that BP roots in Florida are colonized by fewer pathogenic fungi and much more beneficial ones, compared to the *H. patens* native plant. This could be a part of the well-known enemy release phenomenon associated with invasive plants (Rai, 2015; Dawkins and Esiobu, 2016) or possibly resulting from manipulation of soil organisms via root exudates and signaling molecules. Also, the increase in relative abundance of the Alphaproteobacteria in BP correlated with a rise in the prevalence of the Rhizobiales specifically the Rhodoplanes genus and Bradyrhizobiaceae family. These members are essential in nitrogen cycling where Rhodoplanes are members of the denitrifying bacteria and Bradyrhizobiaceae members are able to fix nitrogen (Rodrigues et al., 2015). It was reported that another invasive plant (*Berberis thunbergii* DC) had increased levels of these important microbes involved in nitrogen cycling from the Rhizobiales order (Coats et al., 2014) and could serve a similar beneficial role to the BP.

The singular most distinctive feature of the BP rhizobacteria is found in the members of the phylum *Verrucomicrobia.* In the past, this group was grossly under-represented in previous studies that employed sequencing methods due to PCR-bias (Bergmann et al., 2011). The 515F/806R primer pair used in this study does not exhibit this bias and was able to recover representative populations of this phylum. Little is known about this phylum as most of their members have been uncultured and have mostly been identified by 16S amplicon DNA sequence analysis. The Verrucomicrobia phylum consists of species that are mostly free-living, facultatively anaerobic, and saccharolytic (Bergmann et al., 2011). Compared to the native plants and bulk soil, the Verrucomicrobia community structure of BP had the lowest Shannon’s diversity index but the highest OTU richness of certain unclassified Verrucomicrobia. This phenomenon strongly suggests a possible enrichment strategy in the Verrucomicrobia phylum where only a few species dominate the niche. In this study, the most prevalent was the Chthoniobacteriales order (DA101 genus) from the Spartobacteria class. Under BP rhizosphere, across the sampling sites/replicates there was a significantly higher relative abundance of Verrucomicrobia than the other native plants and bulk soil except again for BP at site 6 where the area was being treated with herbicides and other chemicals unaware to us at the time. The BP plant that was found growing alongside *H. patens* (STHP) did not follow the typical trend of high Verrucomicrobia abundance. This does not disqualify a role and/or possible active colonization at a more mature stage of invasion. To the contrary, it emphasizes the need to evaluate the Verrucomicrobia for potential allelopathic attributes since the BP plant was able to co-exist with the *H. patens* while the Verrucomicrobia population was low. The direct relationship of Verrucomicrobia with plants has been gaining traction as rhizosphere competence was determined in leek (*Allium porrum*) for the *Rhizospheria* Genus (da Rocha et al., 2013). The unknown genus, DA101 from the order Chthoniobacteriales was, however, the most abundant found under BP for the Verrucomicrobia phylum which had been reported as being a very important metabolizer in soil communities (Bergmann et al., 2011). There was a similar trend at the genus level where there was a >50% increase in colonization of DA101 for BP compared to the native plants. One other prominent isolated member of this genus is *Chthoniobacter flavus*, a saccharolytic heterotroph that is able to thrive on plant biomass, but this species was not identified under BP.

This study also showed that the difference in environmental factors at the different sample sites could influence the microbial community structure in the rhizosphere of the invasive plant. At site 6, which had an elevated pH relative to other sites, a different soil physical characteristic and active herbicide treatments, the BP bacterial community structure was different. The prevalence of Verrucomicrobia was diminished under the herbicide-treated BP stands at site 6, while Gammaproteobacteria relative abundance was one of the highest, closely resembling that of native plants. These differences tend to reaffirm the observed BP rhizo-community in other sites as stable and characteristic since the BP stands in site 6 were beginning to wither (unknown to us at sampling time). It is also possible that BP, being a dioecious plant, can exist as many genetic variants (Ferriter, 1997; Williams et al., 2005) with two known varieties (*S. terebinthifolius* var. *raddianus* and var. *terebinthifolius*) thriving in Florida (Ferriter, 1997). There are also two separate haplotypes (A and B) and the more aggressively invasive hybrid BP (Geiger et al., 2011) which could also associate with different microbial communities depending on the exudates each haplotype produces. The genetic variation within species which was not assessed in this study can also significantly affect a plant’s rhizobiome structure (Rai, 2015). This could further explain why only 50% of the BP sequences clustered together on the pCOA plots while sequences from each native plant clustered quite well together at >80% of sites. From the beta diversity pCOA plot (**Figure 3B**), the native plants and bulk soil from most of the sites clustered well together with a significant difference between the different plant communities (*p* = 0.001).

In search of a potential microbial signature of biotic resistance – the ability of resident species to reduce the success of invasive plants (Levine et al., 2004), we plotted the phylotype counts from the invasive plant and the natives from all six locations including the three counties on a Venn diagram (**Figures 4A,B**). The BP rhizosphere had the least number of unique phylotypes in its rhizosphere (766) compared to the *H. patens* and *B. alba* natives (1000 and 874) – **Figure 4A**. This can be indicative of the shorter evolutionary history shared by BP and its exotic range in Florida compared to the two native plants (which have been in Florida much longer) where it associates with less local resident species. Again, BP had the least number of unique phylotypes shared with bulk soil (146) compared to the other natives (222 and 520). This shows a greater disparity between the number of rhizosphere species of BP and the resident species in bulk soil. This larger disparity between BP rhizosphere and bulk soil likely gives BP a better advantage to overcome the ability of the resident bulk soil bacteria to reduce its success. This has not been to our knowledge discussed in any other literature but an important finding that needs further exploration. On a smaller note, *B. alba*, a known Florida native weed, is very common in Florida and grows in a wide variety of geographic locations. It also has a low number of unique phylotypes shared with bulk soil (222) and this helps to strengthen the case of using this information as an indicator of biotic resistance as this plant is considered invasive in China (Tian et al., 2010). *H. patens* which had the highest number of shared phylotypes with bulk soil (520) is mostly used as an ornamental shrub and not readily found in natural areas as common as the other plants; where it’s likely that the resident bulk soil bacteria is able to reduce its success. This unique phylotype data could be indicative of BP’s evolutionary history and ability to overcome biotic resistance.

Acidobacteriales and Burkholderiales were negatively correlated with pH under BP rhizosphere. Relative abundance of members of the order Acidobacteriales from the phylum Acidobacteria were also common under BP soil rhizosphere. Members of the phylum Acidobacteria are another poorly studied group, mostly recovered through culture-independent methods but are very dominant and metabolically active in soil rhizosphere samples (Lee et al., 2008). Acidobacteria form a very diverse phylum, similar to Proteobacteria (Barns et al., 1999) and does have the potential for production of novel antimicrobial compounds and involvement in nitrogen cycling. A correlation was found between Acidobacteriales and Chthoniobacteriales the most prevalent order in Verrucomicrobia (not shown) with an *R*^2^ = 0.9 and *p* = 0.004. The most dominant genus found under the Acidobacteriales order was the Koribacteraceae and an unclassified Ellin6075 genus. The association of Acidobacteriales and Chthoniobacteriales is, however, common and has been experimentally shown to be actively recruited by the leek plant (da Rocha et al., 2013).

## Conclusion

Overall, the OTU richness of bacteria under the BP appears reduced compared to Florida natives but quite intricately associated with specific taxa throughout the different sample sites in its invasive range. The high abundance of members of the Rhodoplanes and Bradyrhizobiaceae from Alphaproteobacteria, the high prevalence of specific members of the Spartobacteria (Verrucomicrobiota) coupled with the low abundance of potentially pathogenic members of the Gammaproteobacteria was a consistent profile of BP root bacteria. These bacteria could play a role in enhancing plant growth, pathogen suppression, and acquisition of nutrients. Taken together, our data suggest that the relative abundance/ratios (not presence or absence) of the rhizobiont groups; including distinctive bacterial taxa are useful signatures of the community structure of the invasive BP rhizobiome. We further underscore the potential value of “shared and unique phylotypes” relative to bulk soil in determining plant colonization/invasion history in a given location. The significance of the high prevalence of Spartobacteria and other genera previously shown to be actively involved in the biology of other plants deserves a close scrutiny as to the putative roles of these organisms during BP invasion.

## Author Contributions

KD contributed to the research, drafting, and editing of the manuscript. NE is the senior author who guided the research, also contributing to research and editing of the manuscript.

## Conflict of Interest Statement

The authors declare that the research was conducted in the absence of any commercial or financial relationships that could be construed as a potential conflict of interest.
