# Supplementary material for: The Invasive Brazilian Pepper Tree (Schinus terebinthifolius) Is Colonized by a Root Microbiome Enriched With Alphaproteobacteria and Unclassified Spartobacteria
Source: Front Microbiol. 2018 May 3;9:876. doi: 10.3389/fmicb.2018.00876 (PMC5943492; doi:10.3389/fmicb.2018.00876)
Supplement: Supplementary file 2 [file Table_2.DOCX]

|  | Df | | Sum Sq | | Mean Sq | | F Value | | Pr (>F) | |
| --- | --- | --- | --- | --- | --- | --- | --- | --- | --- | --- |
| Plant Type | 3 | | 168.74 | | 56.25 | | 8.86 | | 0.00185 | |
| Residuals | 13 | | 82.53 | | 6.35 | |  | |  | |
| Significant plant effect | | Adjusted p value | |  | |  | |  | |  |
| *Schinus*-*Hamelia* | | 0.016 | |  | |  | |  | |  |
| *Schinus-Bidens* | | 0.0121 | |  | |  | |  | |  |

Supplemental Table 2

Table S2: Results of Two Way ANOVA and Tukey HSD post-hoc statistical analysis of the effect of plant type on the prevalence of the Verrucomicrobia phylum
